# Systematic review of pathways to care in the U.S. for Black individuals with early psychosis

**DOI:** 10.1038/s41537-021-00185-w

**Published:** 2021-12-02

**Authors:** Oladunni Oluwoye, Beshaun Davis, Franchesca S. Kuhney, Deidre M. Anglin

**Affiliations:** 1 Elson S. Floyd College of Medicine, Washington State University, Spokane, WA USA; 2 Beth Israel Deaconess Medical Center, Harvard Medical School, Boston, MA USA; 3 Department of Psychology, University of Illinois at Chicago, Chicago, IL USA; 4 Department of Psychology, The City College of New York, New York, NY USA

**Keywords:** Psychosis, Schizophrenia

## Abstract

The pathway to receiving specialty care for first episode psychosis (FEP) among Black youth in the US has received little attention despite documented challenges that negatively impact engagement in care and clinical outcomes. We conducted a systematic review of US-based research, reporting findings related to the pathway experiences of Black individuals with FEP and their family members. A systematic search of PubMed, PsycInfo, and Embase/Medline was performed with no date restrictions up to April 2021. Included studies had samples with at least 75% Black individuals and/or their family members or explicitly examined racial differences. Of the 80 abstracts screened, 28 peer-reviewed articles met the inclusion criteria. Studies were categorized into three categories: premordid and prodromal phase, help-seeking experiences, and the duration of untreated psychosis (DUP). Compounding factors such as trauma, substance use, and structural barriers that occur during the premorbid and prodromal contribute to delays in treatment initiation and highlight the limited use of services for traumatic childhood experiences (e.g., sexual abuse). Studies focused on help-seeking experiences demonstrated the limited use of mental health services and the potentially traumatic entry to services (e.g., law enforcement), which is associated with a longer DUP. Although the majority of studies focused on help-seeking experiences and predictors of DUP, findings suggests that for Black populations, there is a link between trauma and substance use in the pathway to care that impacts the severity of symptoms, initiation of treatment, and DUP. The present review also identifies the need for more representative studies of Black individuals with FEP.

## Introduction

Pathways to care for first episode psychosis (FEP) can be defined as the series of events or contacts with individuals or organizations during the prodromal and onset phase prior to the initiation of outpatient mental health services for FEP^1–3^ and may include contact with primary care, hospitalization, and interactions with local support groups. Moving beyond simply describing help seeking experiences, pathways to care has often been thought of as the time period that aligns with the duration of untreated psychosis (DUP), which is the time period between the onset of delusions or hallucinations and the initiation of treatment^4,5^. However, pathways to mental health care for psychosis does not simply begin at the onset of psychosis, they capture the events and contacts prior to and during the prodromal phase that impact DUP^6^. Structural factors (e.g., neighborhood segregation) differentially distribute access to resources in neighborhoods and communities. This access or lack thereof impacts the length of time spent navigating institutions before individuals with FEP and their families reach the appropriate outpatient mental health services (e.g., coordinated specialty care (CSC)), and the types of experiences encountered^6,7^. There is a clear consensus that understanding pathways to care for underserved individuals is important because the quality of the experiences that precede care initiation impact engagement during treatment and psychiatric and functional outcomes^1^.

Several systematic reviews and meta-analyses have examined pathways to care for FEP, yet did not include research conducted in the US^3,8–10^. For instance, two meta-analyses only included studies conducted in Canada and England. Even though both reviews did not include findings from studies conducted in the US, the authors explored ethnic differences in pathways to mental health services for FEP and found that Black individuals were more likely to have contact with law enforcement and less likely to seek care from a primary physician compared to other racial and ethnic groups^8,9^. Of the other systematic reviews focused on the pathways to care for FEP, one was published more than a decade ago and no reviews distinguished between the unique experiences of Black individuals and their families from those of other racially and ethnically diverse groups^3,11,12^.

Specific to the US, recent studies have identified that Black individuals experiencing FEP are more likely to enter CSC with more severe symptoms and lower quality of life compared to White individuals, and ~80% of Black families had not engaged with a mental health provider^13,14^. These discrepancies highlight Black individuals likely face unique challenges prior to the receipt of CSC or other mental health services. Research in the US tends to focus on identifying racial disparities in access, service utilization, and psychiatric symptoms because it allows researchers, practitioners, and policy makers to explore differences in how services are delivered and implemented. While there are considerable benefits to comparative analyses between racial and ethnic groups, such as identifying differential need, utilizing an emic approach (within-group) focused on the experiences of Black individuals with FEP and their families is imperative to improving their pathways to care and closing the disparities gap earlier^15,16^.

In recent years there has been increased acknowledgment that Black individuals along with their family members experience less desirable pathways to care for FEP, an acknowledgment supported in other countries and demonstrated by the previously described review papers^17^. Building on prior research and given the lack of synthesized findings pertaining to pathways to care for FEP in the US, this study seeks to fill an important gap. The current study aims to systematically assess qualitative, quantitative, and mixed-methods studies that have examined Black individuals’ and/or their family members’ experiences prior to the initiation of outpatient services for FEP in the US.

## Results

### Study characteristics

Twenty-eight articles were included in present review and of these studies, three were comprised of only family members or support persons^18–20^ and four included a mixture of individuals with FEP and their family members^21–24^. The majority of these studies derived from Georgia with ~70% of the studies using data from the Atlanta Cohort on the Early course of Schizophrenia (ACES) project.

Regarding the methodological approach, two (7.1%) articles were categorized as qualitative studies, 25 (89.3%) were quantitative studies, and one (3.6%) study used multiple methods (qualitative and quantitative data methods). Of the two qualitative studies, all used individual interviews with an average sample size of 10 participants. Of the 25 quantitative studies, the majority were cohort studies (24; 96.0%) and the remaining article included baseline data from a clustered randomized controlled trial. The results of this review of pathways to care were divided into three main categories: experiences during the premorbid and prodrome phases, help seeking experiences and treatment delays, and the DUP. A description of study characteristics is presented in Table 1.

**Table 1:** Characteristics of US studies on pathways to care for early psychosis included in review.

Article/Author	Total sample size (*N*)	Demographics (age, sex)	Sample size (%) Black/African American	Location	Methods/procedures
Bergner et al. ^18^ The period of untreated psychosis prior to treatment initiation: A qualitative study of family members’ perspectives	*N* = 12 family member participants	*M*_age_ = 47.8 ± 7.6 years Male: 3 (25.0%)	*N* = 12 (100%)	Georgia	Qualitative—Semi-structured Interviews Data collected between 2004 and 2007
Broussard et al. ^42^ Demographic, socio-environmental, and substance use related predictors of duration of untreated psychosis (DUP)	*N* = 180 participants	*M*_age_ = 24.2 ± 4.9 years Male: 135 (75.0%)	*N* = 154 (85.6%)	Georgia & Washington, DC	Quantitative—Cohort—Correlational
Chien and Compton^36^ The impact of mode of onset of psychosis on pathways to care in a hospitalized, predominantly African-American, first-episode sample	*N* = 76 participants	*M*_age_ = 23.2 ± 4.8 years Male: 59 (77.6%)	*N* = 69 (90.8%)	Georgia	Multiple methods (Quantitative—Cohort—Correlational, Qualitative—semi-structured interviews)
Coleman et al. ^37^ Patterns of health care utilization before first episode psychosis in racial and ethnic groups	*N* = 852 patients	*M*_age_ = 26.9 ± 12.2 years Male: 469 (55%)	*N* = 85 (10.0%)	Southern California, Colorado, Michigan, Minnesota, Washington	Quantitative—Cohort—Descriptive Electronic health records and insurance claims data from 2007 to 2013
Compton et al. ^25^ Preliminary evidence of an association between childhood abuse and cannabis dependence among African American first-episode schizophrenia-spectrum disorder patients	*N* = 18 participants	Age: 50% between 18 and 21 years Male: 16 (88.9%)	*N* = 18 (100%)	Georgia	Quantitative—Cohort—Correlational Data collected between 2002 and 2003
Compton et al. ^38^ A descriptive study of pathways to care among hospitalized urban African American first-episode schizophrenia-spectrum patients	*N* = 25 participants	*M*_age_ = 22.8 ± 4.5 years Male: 19 (76.0%)	*N* = 25 (100%)	Georgia	Quantitative—Cohort—Descriptive Data collected between 2004 and 2005
Compton et al. ^26^ Alcohol and cannabis use in Urban, African American, first-episode schizophrenia-spectrum patients: associations with positive and negative symptoms	*N* = 72 participants	*M*_age_ = 23.4 ± 4.7 years Male: 47 (65.3%)	*N* = 72 (100%)	Georgia	Quantitative—Cohort —Correlational Data collected between 2002 and 2005
Compton et al. ^24^ Mode of onset of psychosis and family involvement in help-seeking as determinants of duration of untreated psychosis	*N* = 73 participants *N* = 35 family member informants	*M*_age_ = 23.5 ± 4.8 years Male: 56 (76.7%)	*N* = 67 (91.8%)	Georgia	Quantitative—Cohort —Correlational Data collected between 2004 and 2007
Compton et al. ^21^ Family-level predictors and correlates of the duration of untreated psychosis in African American first-episode patients	*N* = 42 participants *N* = 42 family member informants	*M*_age_ = 22.1 ± 4.1 years Male: 31 (73.8%) *M*_age_ = 46.3 ± 10.1 years Male: 3 (7.1%)	*N* = 42 (100%)	Georgia	Quantitative—Cohort —Correlational Data collected between 2004 and 2008
Compton et al. ^22^ Health services determinants of the duration of untreated psychosis among African-American first-episode patients	*N* = 42 participants *N* = 42 family member informants	*M*_age_ = 22.1 ± 4.1 years Male: 31 (73.8%) *M*_age_ = 46.3 ± 10.1 years Male: 3 (7.1%)	*N* = 42 (100%)	Georgia	Quantitative—Cohort —Correlational Data collected between 2004 and 2008
Compton et al. ^35^ Characteristics of the retrospectively assessed prodromal period in hospitalized patients with first episode nonaffective psychosis: Findings from a socially disadvantaged, low-income, predominately African American population	*N* = 109 participants	*M*_age_ = 23.1 ± 4.7 years Male: 83 (76.1%)	*N* = 98 (89.9%)	Georgia	Quantitative—Cohort —Descriptive Data collected between 2004 and 2008
Compton et al. ^34^ Subtyping first-episode non-affective psychosis using four early course features: Potentially useful prognostic information at initial presentation	*N* = 200 participants	*M*_age_ = 23.6 ± 4.9 years Male: 145 (72.5%)	*N* = 178 (89.0%)	Georgia	Quantitative—Cohort —Correlational Data collected between 2004 and 2010
Compton et al. ^27^ Abnormal movements in first-episode, nonaffective psychosis: Dyskinesias, stereotypies, and catatonic-like signs	*N* = 47 participants	*M*_age_ = 24.3 ± 5.2 years Male: 33 (70.2%)	*N* = 44 (91.5%)	Unspecified	Quantitative—Cohort —Correlational Data collected between 2008 and 2010
Compton and Esterberg^19^ Treatment delay in first-episode nonaffective psychosis: A pilot study with African American family members and the theory of planned behavior	*N* = 21 family member participants	*M*_age_ = 42.8 ± 10.7 years Male: 7 (33.3%)	*N* = 21 (100%)	Georgia	Quantitative—Cohort —Correlational Data collection period not mentioned
Compton and Furman (2005) Inverse correlations between symptoms scores and spirituality well-being among African American patients with first episode schizophrenia spectrum disorders	*N* = 18 participants	*M*_age_ = 22.3 ± 3.5 years Male: 16 (88.9%)	*N* = 18 (100%)	Georgia	Quantitative—Cohort —Correlational Data collected between 2002 and 2003
Esterberg and Compton^28^ Family history of psychosis negatively impacts age at onset, negative symptoms, and duration of untreated illness and psychosis in first-episode psychosis patients	*N* = 152 participants	*M*_age_ = 22.9 ± 4.5 years Male: 114 (75.0%)	*N* = 136 (89.5%)	Georgia	Quantitative—Cohort —Correlational Data collected between 2004 and 2008
Flanagan and Compton^39^ A comparison of correlates of suicidal ideation prior to initial hospitalization for first‐episode psychosis with prior research on correlates of suicide attempts prior to initial treatment seeking	*N* = 109 participants	*M*_age_ = 23.1 ± 4.7 years Male: 83 (76.1%)	*N* = 98 (89.9%)	Georgia	Quantitative—Cohort —Correlational Data collected between 2004 and 2008
Franz et al. ^20^ Stigma and treatment delay in first-episode psychosis: A grounded theory study	*N* = 12 family member participants	*M*_age_ = 47.8 ± 7.6 years Male: 3 (25%)	*N* = 12 (100%)	Georgia	Qualitative—Semi-structured Interviews Data collected between 2004 and 2007
Goulding et al. ^23^ Family strengths: a potential determinant of the duration of untreated psychosis among hospitalized African‐American first‐episode patients	*N* = 34 participants *N* = 34 family member participants	*M*_age_ = 22.5 ± 4.2 years Male: 23 (68.0%) *M*_age_ = 47.0 ± 11 years Male: 3 (9.0%)	*N* = 34 (100%) *N* = 34 (100%)	Georgia	Quantitative—Cohort —Correlational Data collection period not mentioned
Goulding et al. ^29^ Prevalence and correlates of school drop-out prior to initial treatment of nonaffective psychosis: Further evidence suggesting a need for supported education	*N* = 109 participants	*M*_age_ = 23.1 ± 4.7 years Male: 83 (76.1%)	*N* = 100 (91.7%)	Georgia	Quantitative—Cohort —Descriptive Data collected between 2004 and 2008
Goulding et al. ^40^ Social functioning in urban, predominantly African American, socially disadvantaged patients with first-episode nonaffective psychosis	*N* = 109 participants	*M*_age_ = 23.1 ± 4.7 years Male: 83 (76.1%)	*N* = 100 (91.7%)	Georgia	Quantitative—Cohort —Correlational Data collected between 2004 and 2008
Heun-Johnson et al. ^41^ Association between race/ethnicity and disparities in health care use before first-episode psychosis among privately insured young patients	*N* = 3017 patients	*M*_age_ = 26.9 ± 12.2 years Male: 1534 (50.8%)	*N* = 343 (11.4%)	Nationwide	Quantitative—Cohort —Correlational Medical and prescription drug claims data from 2007 to 2015
Ku et al. ^30^ Neighborhood-level predictors of age at onset and duration of untreated psychosis in first-episode psychotic disorders	*N* = 143 participants	Median_age_ = 22 years Male: 103 (72.0%)	*N* = 123 (86.0%)	Georgia & Washington, DC	Quantitative—Cohort —Correlational Data collected between 2008 and 2013
Langlois et al. ^21^ Adversity in childhood/adolescence and premorbid tobacco, alcohol, and cannabis use among first‐episode psychosis patients	*N* = 247 participants	*M*_age_ = 23.9 ± 4.8 years Male: 184 (74.5%)	*N* = 213 (86.2%)	Georgia & Washington, DC	Quantitative—Cohort —Correlational Data collected between 2008 and 2013
Li et al. ^43^ Longitudinal treatment outcome of African American and Caucasian patients with first episode psychosis	*N* = 199 participants	*M*_age_ = 24.2 ± 7.4 years Male: 44 (65%)	*N* = 62 (31.2%)	Pittsburgh	Quantitative—Cohort Data collected between 1996 and 2004
Nagendra et al. ^13^ Demographic, psychosocial, clinical, and neurocognitive baseline characteristics of Black Americans in the RAISE-ETP study	*N* = 370 participants	*M*_age_ = 23.3 ± 5.11 years Male: 267 (72.2%)	*N* = 152 (56.3%)	Nationwide	Quantitative—Cross-sectional randomized trial—Descriptive Data collected between 2008 and 2012
Ramsay et al. ^32^ Clinical correlates of maltreatment and traumatic experiences in childhood and adolescence among predominantly African American, socially disadvantaged, hospitalized, first-episode psychosis patients	*N* = 61 participants	*M*_age_ = 23.6 ± 5.0 years Male: 44 (72.1%)	*N* = 54 (88.5%)	Georgia	Quantitative—Cohort —Correlational Data collected between 2008 and 2010
Ramsay et al. ^33^ Prevalence and psychosocial correlates of prior incarcerations in an urban, predominantly African-American sample of hospitalized patients with first-episode psychosis	*N* = 109 participants	*M*_age_ = 23.1 ± 4.7 years Male: 83 (76.1%)	*N* = 98 (89.9%)	Georgia	Quantitative—Cohort —Correlational Data collection period not mentioned

### Experiences during the premorbid and prodrome phase

**Fig. 1:**
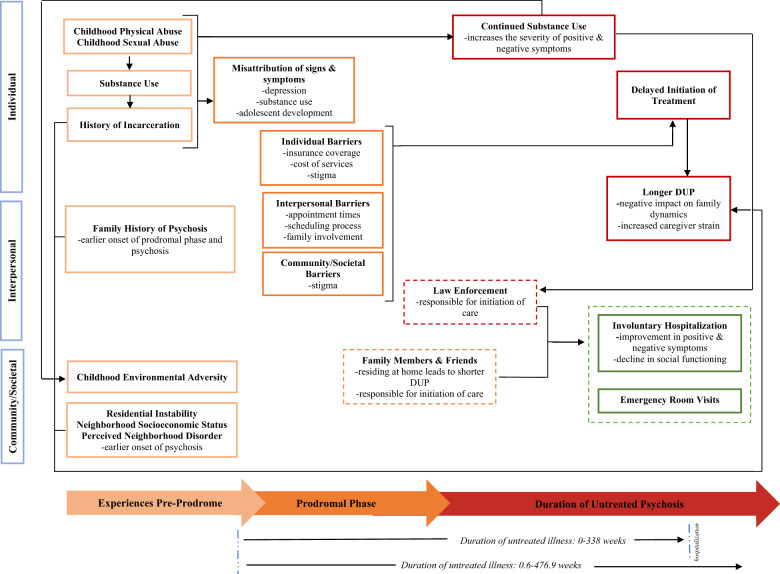
Synthesis of pathways to care for Black individuals with early psychosis. Dashed boxes represent an individual or entity; light orange boxes represent the pre-prodrome phase; orange boxes represent the prodromal phase; red boxes represent experiences during the period of untreated psychosis; green boxes represent contact with services.

Nine studies explored relevant factors associated with age of onset prior to and during the prodromal phase, as illustrated in Fig. 1, and the degree to which substance use and exposure to various traumatic life events occurred during this phase^25–33^. Compton and colleagues demonstrated that among 200 individuals experiencing their FEP, ~90% of whom were Black, more than half met criteria for a substance use disorder^34^ and those with a co-occurring cannabis use disorder were significantly more likely to report childhood sexual and physical abuse compared to those without a cannabis use disorder^25^. Likewise, a larger more recently published study with participants recruited from Georgia and Washington, DC, found that alcohol, cannabis, and tobacco use were associated with exposure to childhood violence and environmental adversity (e.g., exposure to neighborhood violence)^31^. This strongly suggests a link between trauma and substance use in the pathway to care for Black individuals with FEP. Moreover, exposure to childhood traumatic events prior to psychosis onset, a history of incarceration, and leaving high school prematurely were associated with poorer clinical prognostic indicators including severity of positive and negative symptoms^32^ and co-occurring alcohol use or cannabis use disorders at the time of FEP treatment initiation^29,33,34^.

Six studies examined factors associated with duration of the prodromal phase and earlier age of onset of psychosis. Based on three studies from Georgia, the range in the median duration of the prodromal phase was 49–108 weeks^28,34,35^ and the factors associated with age of onset for prodrome or psychosis were captured at multiple levels (from individual to community)^27,28,30^. Esterberg and Compton found that a family history of psychosis (e.g., first degree relative with a non-affective psychotic disorder) was associated with an earlier onset of prodrome and psychosis (16 and 18 years, respectively) compared to those without a family history (age 18 for prodrome; age 22 for psychosis)^28^. Earlier age of onset for psychosis was also associated with neighborhood factors. Ku and colleagues demonstrated, using census data, that the degree of residential instability in one’s neighborhood during adolescence was significantly associated with earlier age of onset even after controlling for possible individual-level confounders (e.g., age at first cannabis use, family history of psychosis, male gender)^30^. At the neighborhood-level, higher residential instability may erode social integration within the local community limiting opportunities for healthy social functioning. Although there is very limited research on the latter, the role of individual- and community-level predictors across the studies included in this review present considerable implications for pathways to the appropriate services.

### Help-seeking experiences and treatment delays

Fourteen studies, including both qualitative and quantitative methods, reported on help-seeking experiences, barriers to treatment, and mental health service utilization among Black individuals and their families^18–21,24,29,32,35–41^. These studies demonstrated that the accumulation of barriers such as financial stress, lack of insurance, lack of affordable mental health services, inflexible appointment times, inefficient scheduling processes, and generally the long process to initiate treatment prolonged the initiation of treatment among Black individuals and their family members (see Fig. 1)^18,19,21,32^.

Two qualitative studies from the ACES cohort and entirely comprised of Black family members recruited from Georgia explored their perspectives on treatment-seeking barriers^18,20^. The qualitative synthesis of these studies suggests that the misattribution of symptoms (e.g., depression, substance use, developmental changes) during the prodromal phase and personal and societal stigma associated with seeking mental health services contributing to delays in treatment^18,20^. One study with 109 study participants recruited from an inpatient hospital located in Georgia, 90% of whom were Black individuals, reported that <15% of participants sought mental health services prior to hospitalization or during the prodromal phase^35^. Findings also noted the initiation of treatment generally occurred after a catalytic event, to which Black individuals with FEP and their family members described as the manifestation of severe positive symptoms (e.g., suicidal or homicidal ideation), interactions with law enforcement, or an accident (e.g., vehicular)^18,20,38,39^.

Two studies reported that family members or friends and law enforcement were primarily responsible for the initiation of treatment or the first hospitalization^36,38^. For instance, Compton and colleagues found that family members and friends made on average four attempts to connect with services prior to the first hospitalization^38^. Notably, initial contact was frequently made with psychiatric or general emergency services, or a mental health professional (e.g., counselor, mental health clinic) and contact with primary care providers were less common^35,36,38^. Perhaps the experiences immediately prior to the onset of psychosis and how Black families make contact with emergency services (e.g., interaction with law enforcement, violent behavior) contributes to the high rate (range: 79–87%) of involuntary hospitalization reported in several studies conducted in Geogria^22,28,32,36,38,39^. Across studies the average age at first hospitalization was 22.9 years (range: 20.9–23.9) which is consistent with findings reported on the average age of onset of psychosis described earlier^13,19,22–24,26,28,31,32,38,39,42^. Furthermore, Black individuals who had dropped out of high school were significantly younger (20.9 ± 3.3 years) at the time of hospitalization compared to those who completed high school (24.9 ± 5.0)^29^. While there are high rates of involuntary hospitalization among Black individuals, one study found that positive and negative symptoms significantly improved six months after hospitalization. However, there were minor decreases in social functioning (e.g., social withdrawal, independence, prosocial activities, employment), which were often present during the prodromal phase^34,40^.

In addition to the results published from the ACES project on Black individuals with FEP and their family members, recent studies utilizing claims data from multiple states have contributed findings on racial and ethnic differences in the utilization of behavioral health services prior to the onset of psychosis^37,41^. These studies demonstrated that Black individuals were significantly less likely to use outpatient mental health services, and significantly more likely to visit the emergency room in the year prior to the onset of psychosis, relative to non-Hispanic White individuals. The underutilization of behavioral health services among Black individuals prior to FEP may be a plausible explanation for the decreased likelihood that Black individuals will have a comorbid diagnosis prior to onset of psychosis^37,41^. However, this also demonstrates the limited use of behavioral health services among Black individuals for signs and symptoms (e.g., substance use, physical and sexual abuse) or experiences that occurred prior to the onset of psychosis.

### Duration of untreated psychosis

Ten studies with participants comprised predominately (86–100%) of Black individuals with FEP or their family members reported findings relevant to the DUP^13,21–24,28,30,36,42,43^.

Figure 1 displays the summative findings from four studies conducted primarily in Georgia which identified individual-level factors (e.g., lack of insurance, financial strain, incarceration, substance use, chronic mode of onset) that were associated with a longer DUP^21,24,36,42^. For example, in a subsample involving 42 Black individuals hospitalized with FEP derived from the ACES project, those without health insurance, experiencing financial issues, or experiencing four or more additional barriers (e.g., transportation problems, conflict with work schedule) had a significantly longer DUP, than those with insurance, who were financially stable, and experienced none or one barrier^22^. Three of these studies further explored mode of onset, defined as the speed with which positive symptoms emerge, and its association with DUP. One study found that a chronic mode of onset for psychosis was associated with a longer DUP compared to an acute mode of onset for psychosis^24^. One of the other studies found those with a more chronic onset had a significantly longer duration to first help-seeking contact relative to an acute mode of onset and found no differences in the time from first-help seeking contact to hospitalization^36^.

Five studies, primarily conducted in Georgia, identified the association between DUP and interpersonal-level factors focused on family characteristics^22–24,28,42^. In a predominately (~90%) Black sample of participants, a family history of psychosis was associated with a significantly longer DUP compared to those without a family history^28^. Interestingly, family involvement in help-seeking was associated with a longer DUP^24^. Yet, Black individuals residing with a family member was predictive of a shorter DUP compared to those residing with other individuals or alone^42^. Taken together with the qualitative findings reported earlier, it may be that the misattribution of symptoms and stigma associated with psychosis contributes to the delaying the initiation of treatment, thus lengthening the DUP. In addition to certain family-level characteristics lengthening the DUP among Black individuals with FEP, findings from two studies demonstrated that longer DUP had a negative impact on family strengths (i.e., shared values and beliefs, expression of feelings, conflicts, reoccurring problems) and was associated with increased caregiver strain^22,23^. Ku and colleagues conducted one of the only studies to examine community-level characteristics in Georgia and Washington, DC, specifically general neighborhood socioeconomic status and perceived neighborhood disorder, which correlated with a longer DUP^30^.

Only two studies, one conducted in Pittsburg and the other using a more nationally representing sample, examined whether there were significant differences in the DUP between Black and White individuals with FEP^13,43^. While both studies reported an extended DUP for Black individuals relative to White individuals (Nagendra: 89 weeks vs. 70 weeks; Li: 2.47 years vs. 1.82 years), no statistically significant differences were noted^13,43^.

## Discussion

The 28 reviewed studies focused on the pathways to care for Black individuals with FEP and their families emanate from a relatively small group of data collection sites (e.g., Georgia mental health care system) and with a restricted range sociodemographically (i.e., no to low income). As such, there is a clear need for more representative studies of Black individuals with FEP across a wider range of socioeconomic characteristics. Nevertheless, as illustrated in Fig. 1, the results of the present review revealed salient experiences during the premorbid and prodromal phases of the pathway to care for Black individuals with FEP and extends previous work done on Black populations in Canada and the UK which mainly found police involvement was higher among Black individuals in their pathway to care compared to White individuals^8,9^. The synthesis of findings across nine studies suggest the premorbid and prodromal phases tend to be characterized by exposure to traumatic experiences or negative life events, including childhood adversity, history of incarceration and neighborhood violence; and that these exposures are associated with increased likelihood of substance abuse^25–33^. Moreover, four studies identified similar factors (e.g., incarceration, substance use, neighborhood disorder) were related to longer DUP. Substance use may be a way young Black individuals are coping with trauma experienced at both the individual and neighborhood levels; a pattern found in previous research^29,32–34^.

The presence of substance use during the prodromal phase may be connected to why Black family members in a qualitative study indicated a tendency to misattribute symptoms to substance use effects^18^. Findings from two studies that utilized claims data found Black individuals were also less likely to have engaged behavioral mental health services for these negative life event exposures that precede onset of psychotic symptoms^37,41^. This may be connected to findings from two qualitative family studies which found societal stigma about seeking mental health services or structural barriers such as financial strains and limited access were reported by a number of Black individuals and families^18,20^. Stigmatizing attitudes about people with mental illness and racial stereotypes about Black people both entail perceptions of dangerousness—a perception that could and has led to dire consequences for Black individuals.^44^ Some of the hesitancy to engage mental healthcare systems is likely connected to a fear of being perceived and consequentially treated as a threat—a valid concern given the disproportionate number of Black individuals with mental illness housed in jails^45^. This combination of factors (stigma, structural barriers, misattribution of symptoms) contributes to more delays in treatment initiation—delays that may connect to why Black individuals have been more likely to present with more severe psychotic symptoms^13^. Black families reported that it often took some catalytic event to force their loved ones into treatment^18,20^. Given the police surveillance bias prevalent in Black communities^46^, the catalytic event is more likely to involve law enforcement^47^, especially because of the acuity and severity of psychotic symptoms more likely to be reported prior to initiating early psychosis treatment^13^. This challenging path contributes to delays in the initiation of treatment for Black families, thus lengthening the DUP.

While findings from two studies found no significant difference in the DUP between Black and White individuals, both studies found the duration was longer in Black individuals by as much as eight months. Furthermore, findings from two studies suggest longer DUP contributes to the wear and tear on family functioning and increases strain among caregivers in particular^23,24^. Black families that are already dealing with structural barriers (e.g., insurance, accessibility) have to also deal with a frustrating drawn out process when attempts are made to initiate treatment^18,19,21,32^. Structural barriers are directly connected to a history of structural racism and racialized capitalism in the US^48^, whereby quality healthcare access is often tied to social capital unequally distributed in racially segregated societies^49^. Black families with a history of psychosis likely experience these barriers and strains even more, contributing to an even longer DUP among individuals experiencing their FEP. The social drift hypothesis of psychosis postulates that individuals with psychosis experience a decline in socioeconomic functioning due to the ramifications of illness experienced over time across generations^50,51^. This may make mobilizing resources for that second generation of affected offspring dealing with early psychosis more challenging, especially in an inequitable healthcare system with a long history of discriminating against Black people^52^.

Treatment delays are common among individuals with early psychosis regardless of race, however, the pathway to get there has been identified as more traumatic and less straightforward for Black individuals^53^. Results from this review complement an earlier report on pathways which described stigma, beliefs about causes of mental illness, and lower probability to be referred for psychiatric care by general practitioners, as factors related to treatment delays for African Americans with early psychosis^17^. One glaring omission from that earlier report which continues in the current review is the lack of studies explicitly examining systemic racism and discrimination, and their connection to the factors identified in this review (e.g., age of onset, DUP, barriers, stigma, substance use, trauma). Racism has not been studied at the individual or structural level among Black individuals with early psychosis even though racism is a historical system of oppression that especially shapes social determinants of psychosis for Black people^54^, as well as the probability of having traumatizing entry points into mental healthcare systems (e.g., law enforcement, involuntary hospital admissions)^28,35,36,38^. How might dismantling the systems structured through a racialized hierarchy where Black individuals represent the most distal category^55,56^, improve the pathway to care in ways that also improve engagement and treatment outcomes? A few studies included in this review revealed neighborhood-level community factors were relevant in the pathway to care for Black individuals because they were related to two prognostic indicators—residential instability in neighborhoods was related to earlier age of psychosis onset and low neighborhood socioeconomic status and perceived neighborhood disorder were related to longer DUP. This represents a first step in identifying macro-level structural factors that influence pathways to care for Black individuals. More research in this area is needed.

This review provides valuable insight into the complexity of why pathways to care are so challenging for Black individuals and their families by specifying important factors during more than just immediate entrance into treatment. The current review was inclusive of premorbid and prodromal phases of the pathway as well as help-seeking behaviors. In that vein, we have connected findings from literatures along this path in meaningful ways that can be explored in future studies. Nevertheless, certain limitations should be noted. First, the quality appraisal for studies included in the systematic review was not used as basis for inclusion criteria but to highlight any methodological limitations, such as the adequate representation of Black/African American participants in studies. Relatedly, studies either used self-reported race and ethnicity or obtained race and ethnicity information from a chart review based on a clinician’s assessment, which could result in some participants in these studies being misclassified as Black. Nevertheless, the majority of studies came from a mental health system in Georgia that serves a predominantly Black community, and any misclassification would likely be negligible. Second, the present study focused on pathways to care, which can be considered a broad topic of focus, however; this review demonstrates specific areas for further research. Additionally, the present study was necessarily focused on the experiences of Black/African American of mental health services, so conclusions are framed largely within the context of the American experience. Despite preliminary evidence of differences in treatment seeking experiences among various ethnicities and nationalities (e.g., Black, African, Caribbean, etc.) across the diaspora of the Black community evident by studies conducted in Europe^8,9^, the racialized historical context in the US, is strongly anti-Black regardless of immediate ancestry^46,55^. Nevertheless, more research that captures cultural heterogeneity within the Black community that characterizing pathways to care, DUP, and barriers to treatment initiation should be conducted in the future. Moreover, studies included in this review did not account for other important differences among Black people including regional, socioeconomic, and immigration-related differences. Future analyses should seek to explore within group differences for different socioeconomic and ethnic subgroups within the Black American experience.

This review fills an important gap in the literature by focusing on Black populations specifically and by including studies that gather data from Black family members using a mixture of methods (e.g., qualitative). As synthesized and summarized in Fig. 1, it reveals more specific elements along the pathway that can be targeted in community outreach efforts in early intervention and prevention, and in future studies. From targeting stigma with a culturally tailored lens^12^ to dismantling structural barriers and improving access to behavioral interventions for coping with trauma and racialized trauma specifically, these findings identify potential targets for prevention and community outreach to disrupt these deleterious pathways that leave Black individuals starting CSC already at a disadvantage^13^, and perhaps why some Black individuals are not accessing CSC programs due to ineligibility (e.g., substance use, long DUP > 2 years). There is considerable evidence that the need to go upstream and dismantle the policies shaped by structural racism is a place for future emphasis with more focus on the factors that precede onset of acute psychotic symptoms^54,57^. We have synthesized findings that identify several related factors that could impact treatment engagement prior to treatment initiation. For example, in Black populations, the unfortunate contact with law enforcement at the entry point of a medical system (e.g., being brought to ER via police) is usually what gets emphasized for pathways to care among Black individuals. However, our review suggests contact with law enforcement is happening during premorbid and prodromal phases in a way that is linked with the substance use being used to cope with trauma, as well as the exacerbation of the psychotic symptoms themselves. Opportunities to disrupt these pathways to care exist for Black individuals with FEP in the US and will require more prevention efforts and structural change.

## Methods

### Search strategies

The present study was guided by the Reporting Items for Systematic Reviews and Meta-Analyses (PRISMA) using a two-step literature search approach^58^. It should be noted that the present study was not registered with the International Prospective Register of Systematic Reviews (PROSPERO) but the authors ensured that no duplicate review had been published or is currently being performed. In step one, the search to retrieve studies was performed in three electronic databases, PubMed, PsycInfo, and Embase/Medline, with no date restrictions. The initial literature search was performed in October 2020 and updated in April 2021. The following search terms along with Boolean operators were used to increase sensitivity of the search strategy: (“first episode psychosis” OR “early psychosis” OR “first episode schizophrenia”) AND (“African American” OR “Black” OR “ethnic disparities” OR “racial disparities”) AND (“United States” OR “US” OR America”). As shown in Fig. 1, the search results from PubMed resulted in 44 articles, PsycInfo resulted in 35 articles, and Embase/MEDLINE resulted in 48 articles. Step two identified additional studies by hand-searching the reference lists of identified studies, which resulted in nine articles. Search results were imported into Covidence (www.covidence.org; Veritas Health Innovation Ltd.), a web-based tool developed to assist in the screening and organization of systematic reviews^59^.

### Selection criteria

**Fig. 2:**
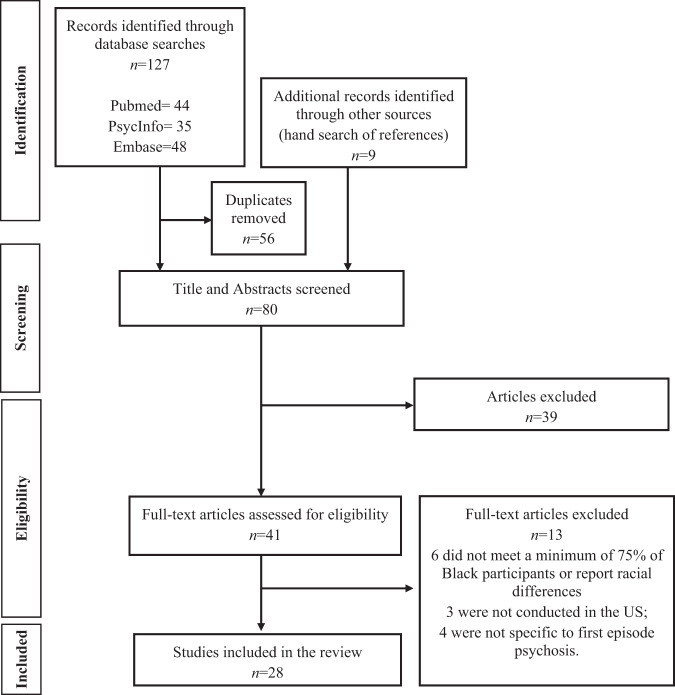
Study selection flow for systematic reviews (PRISMA).

Studies were included if the following criteria were met: (1) at least 75% of sample was Black/African American and/or specific findings on racial differences focused on Black/African Americans participants, consistent with prior reviews focused on Black/African American individuals and behavioral health interventions^60,61^; (2) a population focus on individuals experiencing their first episode of affective or non-affective psychosis and/or their family members/support persons; (3) qualitative, quantitative, or mixed method studies focused on understanding experiences prior to receipt of outpatient services for FEP; (4) studies published in English; and (5) studies published in peer-reviewed journals. Studies were excluded if they did not meet any of the above criteria, as well as the following exclusion criteria: (1) not conducted in the US; (2) if methods did not clearly define the sample as a first episode; and (3) abstracts, systematic reviews or meta-analyses, case-reports, case studies, thesis and dissertations, and gray literature. The initial screening of titles and abstracts (*n* = 80) was performed by one author (O.O.). Two authors (O.O. and D.M.A.) read full-text articles independently for study inclusion. Six studies did not reach consensus based on independent reviews and were resolved by discussion, resulting in the exclusion of five studies and a final sample of 28 studies (see Fig. 2 for details).

### Data extraction

Data from the 28 included studies were extracted into an extraction table which included data on participant demographics (sample size, age, sex, and the number of Black/African American participants), geographic location of study setting, study design, and major findings.

### Quality assessment

The methodological quality of each study was assessed using the validated mixed methods appraisal tool (MMAT). The MMAT has been used in prior systematic reviews and is a checklist comprised of 27-items used to assess the quality of qualitative, quantitative, and mixed methods studies included in systematic reviews^62–66^. Items 1–2 on the MMAT are general screening questions. Items 1.1.−1.5. are used to assess qualitative studies, items 2.1.−2.5. are used to assess quantitative randomized controlled studies, items 3.1.−3.5. are used to assess quantitative non-randomized studies, items 4.1.−4.5. are used to assess quantitative descriptive studies, and items 5.1.−5.5. are used to assess mixed methods studies. The overall quality of a study is presented as a percentage, with higher values reflecting greater quality. Two authors (O.O. and D.M.A.) independently rated each study using the MMAT and met to discuss the methodological quality of each study. Based on independent ratings and discussion, 19 studies were rated medium (60%), nine were rated high (80–100%) quality, and no studies were excluded (see Supplementary Table 1).

### Reporting summary

Further information on research design is available in the Nature Research Reporting Summary linked to this article.

## Supplementary information

Reporting Summary

Supplementary Information

## Data Availability

This is a systematic review and data sharing is not applicable to this article. Data generated and analyzed during this study have been published in the present article or provided as supplementary materials.

## References

[1] MacDonald K, Fainman-Adelman N, Anderson KK, Iyer SN (2018). Pathways to mental health services for young people: a systematic review. Soc. Psychiatry Psychiatr. Epidemiol..

[2] Rogler LH, Cortes DE (1993). Help-seeking pathways: a unifying concept in mental health care. Am. J. Psychiatry.

[3] Singh SP, Grange T (2006). Measuring pathways to care in first-episode psychosis: a systematic review. Schizophr. Res..

[4] Addington J (2015). Duration of untreated psychosis in community treatment settings in the United States. Psychiatr. Serv. Wash. DC.

[5] Cabassa LJ (2018). Understanding pathways to care of individuals entering a specialized early intervention service for first-episode psychosis. Psychiatr. Serv. Wash. DC.

[6] Alvidrez J, Castille D, Laude-Sharp M, Rosario A, Tabor D (2019). The National Institute on Minority Health and Health Disparities research framework. Am. J. Public Health.

[7] Williams DR, Williams-Morris R (2000). Racism and mental health: the African American experience. Ethn. Health.

[8] Anderson KK, Flora N, Archie S, Morgan C, McKenzie K (2014). A meta-analysis of ethnic differences in pathways to care at the first episode of psychosis. Acta Psychiatr. Scand..

[9] Halvorsrud K, Nazroo J, Otis M, Brown Hajdukova E, Bhui K (2018). Ethnic inequalities and pathways to care in psychosis in England: a systematic review and meta-analysis. BMC Med..

[10] Lilford P, Wickramaseckara Rajapakshe OB, Singh SP (2020). A systematic review of care pathways for psychosis in low-and middle-income countries. Asian J. Psychiatry.

[11] Anderson KK, Fuhrer R, Malla AK (2010). The pathways to mental health care of first-episode psychosis patients: a systematic review. Psychol. Med..

[12] Gronholm PC, Thornicroft G, Laurens KR, Evans-Lacko S (2017). Mental health-related stigma and pathways to care for people at risk of psychotic disorders or experiencing first-episode psychosis: a systematic review. Psychol. Med..

[13] Nagendra A (2018). Demographic, psychosocial, clinical, and neurocognitive baseline characteristics of Black Americans in the RAISE-ETP study. Schizophr. Res..

[14] Oluwoye, O. et al. The impact of early family contact on quality of life among non-Hispanic Blacks and Whites in the RAISE-ETP trial. *Schizophr. Res*. 10.1016/j.schres.2019.12.004 (2020). 10.1016/j.schres.2019.12.004 PMC7239728 31902559

[15] Bediako, S. M. & Griffith, D. M. Eliminating racial/ethnic health disparities: reconsidering comparative approaches. *J Health Dispar Res Pract.*10.13016/m2ksmy-m5v1 (2020).

[16] Singh, S. P. et al. *Ethnicity, Detention and Early Intervention: Reducing Inequalities and Improving Outcomes for Black and Minority Ethnic Patients: The Enrich Programme, a Mixed-methods Study. Ethnicity, Detention and Early Intervention: Reducing Inequalities and Improving Outcomes for Black and Minority Ethnic Patients: The ENRICH Programme, a Mixed-methods Study* (NIHR Journals Library, 2013). 27466650

[17] Merritt-Davis OB, Keshavan MS (2006). Pathways to care for African Americans with early psychosis. Psychiatr. Serv. Wash. DC.

[18] Bergner E (2008). The period of untreated psychosis before treatment initiation:: a qualitative study of family members’ perspectives. Compr. Psychiatry.

[19] Compton MT, Esterberg ML (2005). Treatment delay in first-episode nonaffective psychosis: a pilot study with African American family members and the theory of planned behavior. Compr. Psychiatry.

[20] Franz L (2010). Stigma and treatment delay in first‐episode psychosis: a grounded theory study. Early Interv. Psychiatry.

[21] Compton MT (2009). Health services determinants of the duration of untreated psychosis among African-American first-episode patients. Psychiatr. Serv. Wash. DC.

[22] Compton MT, Goulding SM, Gordon TL, Weiss PS, Kaslow NJ (2009). Family-level predictors and correlates of the duration of untreated psychosis in African American first-episode patients. Schizophr. Res..

[23] Goulding SM (2008). Family strengths: a potential determinant of the duration of untreated psychosis among hospitalized African-American first-episode patients. Early Interv. Psychiatry.

[24] Compton MT, Chien VH, Leiner AS, Goulding SM, Weiss PS (2008). Mode of onset of psychosis and family involvement in help-seeking as determinants of duration of untreated psychosis. Soc. Psychiatry Psychiatr. Epidemiol..

[25] Compton MT, Furman AC, Kaslow NJ (2004). Preliminary evidence of an association between childhood abuse and cannabis dependence among African American first-episode schizophrenia-spectrum disorder patients. Drug Alcohol Depend..

[26] Compton MT, Whicker NE, Hochman KM (2007). Alcohol and cannabis use in urban, African American, first-episode schizophrenia-spectrum patients: associations with positive and negative symptoms. J. Clin. Psychiatry.

[27] Compton MT, Fantes F, Wan CR, Johnson S, Walker EF (2015). Abnormal movements in first-episode, nonaffective psychosis: dyskinesias, stereotypies, and catatonic-like signs. Psychiatry Res..

[28] Esterberg M, Compton M (2012). Family history of psychosis negatively impacts age at onset, negative symptoms, and duration of untreated illness and psychosis in first-episode psychosis patients. Psychiatry Res..

[29] Goulding SM, Chien VH, Compton MT (2010). Prevalence and correlates of school drop-out prior to initial treatment of nonaffective psychosis: further evidence suggesting a need for supported education. Schizophr. Res..

[30] Ku, B. S., Pauselli, L., Manseau, M. & Compton, M. T. Neighborhood-level predictors of age at onset and duration of untreated psychosis in first-episode psychotic disorders. *Schizophr. Res*. 10.1016/j.schres.2019.12.036 (2020). 10.1016/j.schres.2019.12.036 PMC7299734 31948900

[31] Langlois, S., Zern, A., Kelley, M. E. & Compton, M. T. Adversity in childhood/adolescence and premorbid tobacco, alcohol, and cannabis use among first-episode psychosis patients. *Early Interv. Psychiatry***n/a**, (2020). 10.1111/eip.13086 33289325

[32] Ramsay CE, Flanagan P, Gantt S, Broussard B, Compton MT (2011). Clinical correlates of maltreatment and traumatic experiences in childhood and adolescence among predominantly African American, socially disadvantaged, hospitalized, first-episode psychosis patients. Psychiatry Res..

[33] Ramsay CE (2011). Prevalence and psychosocial correlates of prior incarcerations in an urban, predominantly African-American sample of hospitalized patients with first-episode psychosis. J. Am. Acad. Psychiatry Law.

[34] Compton MT, Kelley ME, Ionescu DF (2014). Subtyping first-episode non-affective psychosis using four early-course features: potentially useful prognostic information at initial presentation. Early Interv. Psychiatry.

[35] Compton MT, Goulding SM, Walker EF (2010). Characteristics of the retrospectively assessed prodromal period in hospitalized patients with first-episode nonaffective psychosis: findings from a socially disadvantaged, low-income, predominantly African American population. J. Clin. Psychiatry.

[36] Chien VH, Compton MT (2008). The impact of mode of onset of psychosis on pathways to care in a hospitalized, predominantly African-American, first-episode sample. *Early Interv*. Psychiatry.

[37] Coleman KJ (2019). Patterns of health care utilization before first episode psychosis in racial and ethnic groups. Ethn. Dis..

[38] Compton MT, Esterberg ML, Druss BG, Walker EF, Kaslow NJ (2006). A descriptive study of pathways to care among hospitalized urban African American first-episode schizophrenia-spectrum patients. Soc. Psychiatry Psychiatr. Epidemiol..

[39] Flanagan P, Compton MT (2012). A comparison of correlates of suicidal ideation prior to initial hospitalization for first-episode psychosis with prior research on correlates of suicide attempts prior to initial treatment-seeking. Early Interv. Psychiatry.

[40] Goulding SM, Franz L, Bergner E, Compton MT (2010). Social functioning in urban, predominantly African American, socially disadvantaged patients with first-episode nonaffective psychosis. Schizophr. Res..

[41] Heun-Johnson H (2021). Association between race/ethnicity and disparities in health care use before first-episode psychosis among privately insured young patients. JAMA Psychiatry.

[42] Broussard B (2013). Demographic, socio-environmental, and substance-related predictors of duration of untreated psychosis (DUP). Schizophr. Res..

[43] Li H, Eack SM, Montrose DM, Miewald JM, Keshavan M (2011). Longitudinal treatment outcome of African American and Caucasian patients with first episode psychosis. Asian J. Psychiatry.

[44] Anglin DM, Link BG, Phelan JC (2006). Racial differences in stigmatizing attitudes toward people with mental illness. Psychiatr. Serv..

[45] Appel O, Stephens D, Shadravan SM, Key J, Ochoa K (2020). Differential incarceration by race-ethnicity and mental health service status in the Los Angeles County Jail System. Psychiatr. Serv..

[46] Sewell W, Horsford CE, Coleman K, Watkins CS (2016). Vile vigilance: an integrated theoretical framework for understanding the state of Black surveillance. J. Hum. Behav. Soc. Environ..

[47] Cha-Jua SK (2014). We believe it was murder. Black Sch..

[48] Laster Pirtle WN (2020). Racial capitalism: a fundamental cause of novel coronavirus (COVID-19) pandemic inequities in the United States. Health Educ. Behav..

[49] Lee M-A (2009). Neighborhood residential segregation and mental health: a multilevel analysis on Hispanic Americans in Chicago. Soc. Sci. Med..

[50] Dohrenwend BP (1992). Socioeconomic status and psychiatric disorders: the causation-selection issue. Science.

[51] Goldberg EM, Morrison SL (1963). Schizophrenia and social class. Br. J. Psychiatry.

[52] Gee GC, Ford CL (2011). Structural racism and health inequities. Bois Rev. Soc. Sci. Res. Race.

[53] Commander MJ, Cochrane R, Sashidharan SP, Akilu F, Wildsmith E (1999). Mental health care for Asian, black and white patients with non-affective psychoses: pathways to the psychiatric hospital, in-patient and after-care. Soc. Psychiatry Psychiatr. Epidemiol..

[54] Anglin, D. M. et al. From womb to neighborhood: a racial analysis of social determinants of psychosis in the United States. *Am. J. Psychiatry.*10.1176/appi.ajp.2020.20071091 (2021). 10.1176/appi.ajp.2020.20071091 PMC8655820 33934608

[55] Bobo LD (2011). Somewhere between Jim Crow & Post-Racialism: reflections on the racial divide in America Today. Daedalus.

[56] Gold SJ (2004). From Jim Crow to racial hegemony: evolving explanations of racial hierarchy. Ethn. Racial Stud..

[57] Anglin, D. M., Galea, S. & Bachman, P. Going upstream to advance psychosis prevention and improve public health. *JAMA Psychiatry.*10.1001/jamapsychiatry.2020.0142 (2021). 10.1001/jamapsychiatry.2020.0142 32236511

[58] Moher D, Liberati A, Tetzlaff J, Altman DG (2009). Preferred reporting items for systematic reviews and meta-analyses: the PRISMA statement. Ann. Intern. Med..

[59] Harrison H, Griffin SJ, Kuhn I, Usher-Smith JA (2020). Software tools to support title and abstract screening for systematic reviews in healthcare: an evaluation. BMC Med. Res. Methodol..

[60] Montgomery L, Burlew AK, Haeny AM, Jones CA (2020). A systematic scoping review of research on Black participants in the National Drug Abuse Treatment Clinical Trials network. Psychol. Addict. Behav. J. Soc. Psychol. Addict. Behav..

[61] Sutton MY, Lasswell SM, Lanier Y, Miller KS (2014). Impact of parent–child communication interventions on sex behaviors and cognitive outcomes for Black/African-American and Hispanic/Latino Youth: a systematic review, 1988–2012. J. Adolesc. Health . Publ. Soc. Adolesc. Med..

[62] Ferrie J, Miller H, Hunter SC (2020). Psychosocial outcomes of mental illness stigma in children and adolescents: a mixed-methods systematic review. Child. Youth Serv. Rev..

[63] Hong QN (2018). The Mixed Methods Appraisal Tool (MMAT) version 2018 for information professionals and researchers. Educ. Inf..

[64] Jordan G (2017). Positive changes experienced after a first episode of psychosis: a systematic review. Psychiatr. Serv..

[65] Pluye P (2012). Critical appraisal tools for assessing the methodological quality of qualitative, quantitative and mixed methods studies included in systematic mixed studies reviews. J. Eval. Clin. Pract..

[66] Souto RQ (2015). Systematic mixed studies reviews: updating results on the reliability and efficiency of the Mixed Methods Appraisal Tool. Int. J. Nurs. Stud..

