# Supplementary material for: Systematic review of pathways to care in the U.S. for Black individuals with early psychosis
Source: NPJ Schizophr. 2021 Dec 2;7:58. doi: 10.1038/s41537-021-00185-w (PMC8639758; doi:10.1038/s41537-021-00185-w)
Supplement: Supplementary file 2 — Supplementary Information [file 41537_2021_185_MOESM2_ESM.pdf]

Supplement Table 1. MMAT quality appraisal summary

| Article/Author | | Criterion | | | | | | |
|---|---|---|---|---|---|---|---|---|
| | | Study Type | 1 | 2 | 3 | 4 | 5 | Qualitative percentage (%) |
| 1 | Bergner et al., 2008<br>The period of untreated psychosis prior to treatment initiation: A qualitative study of family members' perspectives | Qualitative | Y | Y | Y | Y | Y | 100% |
| 2 | Broussard et al., 2013<br>Demographic, socio-environmental, and substance use related predictors of duration of untreated psychosis (DUP). | Quantitative Descriptive | Y | N | Y | Y | Y | 80% |
| 3 | Chien & Compton., 2008<br>The impact of mode of onset of psychosis on pathways to care in a hospitalized, predominantly African-American, first-episode sample. | Quantitative Descriptive | Y | Y | Y | N | Y | 80% |
| 4 | Coleman et al., 2019<br>Patterns of health care utilization before first episode psychosis in racial and ethnic groups. | Quantitative Descriptive | Y | Y | N | Y | Y | 80% |
| 5 | Compton et al., 2004. Preliminary evidence of an association between childhood abuse and cannabis dependence among African American first-episode schizophrenia-spectrum disorder patients. | Quantitative Descriptive | Y | N | Y | N | Y | 60% |
| 6 | Compton & Esterberg, 2005<br>Treatment delay in first-episode nonaffective psychosis: A pilot study with African American family members and the theory of planned behavior. | Quantitative Descriptive | Y | N | Y | N | Y | 60% |
| 7 | Compton et al., 2005<br>Inverse correlations between symptoms scores and spirituality well-being among African American patients with first episode schizophrenia spectrum disorders. | Quantitative Descriptive | Y | N | Y | N | Y | 60% |
| 8 | Compton et al., 2006<br>A descriptive study of pathways to care among hospitalized urban African American first-episode schizophrenia-spectrum patients | Quantitative Descriptive | Y | Y | Y | - | Y | 80% |
| 9 | Compton et al., 2007.<br>Alcohol and cannabis use in Urban, African American, first-episode schizophrenia-spectrum patients: associations with positive and negative symptoms. | Quantitative Descriptive | Y | N | N | Y | Y | 60% |
| 10 | Compton et al., 2008<br>Mode of onset of psychosis and family involvement in help-seeking as determinants of duration of untreated psychosis. | Quantitative Descriptive | Y | Y | Y | N | Y | 80% |
| 11 | Compton et al., 2009<br>Health services determinants of the duration of untreated psychosis among African-American first-episode patients. | Quantitative Descriptive | Y | N | Y | N | Y | 60% |
| 12 | Compton et al., 2009<br>Family-level predictors and correlates of the duration of untreated psychosis in African American first-episode patients. | Quantitative Descriptive | Y | N | Y | N | Y | 60% |
| 13 | Compton et al., 2010<br>Characteristics of the retrospectively assessed prodromal period in hospitalized patients with first episode nonaffective psychosis: Findings from a socially disadvantaged, low-income, predominately African American population. | Quantitative Descriptive | Y | N | Y | N | Y | 60% |
| 14 | Compton et al., 2014.<br>Subtyping first-episode non-affective psychosis using four early course features: Potentially useful prognostic information at initial presentation | Quantitative Descriptive | Y | N | Y | N | Y | 60% |

| | | | | | | | |
|---|---|---|---|---|---|---|---|
| 15 | Compton et al., 2015. Abnormal movements in first-episode, nonaffective psychosis: Dyskinesias, stereotypies, and catatonic-like signs. | Quantitative Descriptive | Y | N | Y | N | Y | 60% |
| 16 | Esterberg & Compton, 2012<br>Family history of psychosis negatively impacts age at onset, negative symptoms, and duration of untreated illness and psychosis in first-episode psychosis patients. | Quantitative Descriptive | Y | N | Y | N | Y | 60% |
| 17 | Flanagan et al., 2012.<br>A comparison of correlates of suicidal ideation prior to initial hospitalization for first□episode psychosis with prior research on correlates of suicide attempts prior to initial treatment seeking. | Quantitative Descriptive | Y | N | Y | N | Y | 60% |
| 18 | Franz et al., 2010<br>Stigma and treatment delay in first-episode psychosis: A grounded theory study. | Qualitative | Y | Y | Y | Y | Y | 100% |
| 19 | Goulding et al., 2008<br>Family strengths: a potential determinant of the duration of untreated psychosis among hospitalized African□American first□episode patients. | Quantitative Descriptive | Y | N | Y | N | Y | 60% |
| 20 | Goulding et al., 2010<br>Social functioning in urban, predominantly African American, socially disadvantaged patients with first-episode nonaffective psychosis. | Quantitative Descriptive | Y | N | Y | N | Y | 60% |
| 21 | Goulding et al., 2010.<br>Prevalence and correlates of school drop-out prior to initial treatment of nonaffective psychosis: Further evidence suggesting a need for supported education. | Quantitative Descriptive | Y | N | Y | N | Y | 60% |
| 22 | Heun-Johnson et al., 2021<br>Association between race/ethnicity and disparities in health care use before first-episode psychosis among privately insured young patients. | Quantitative Descriptive | Y | Y | Y | N | Y | 80% |
| 23 | Ku et al., 2020<br>Neighborhood-level predictors of age at onset and duration of untreated psychosis in first-episode psychotic disorders. | Quantitative Descriptive | Y | N | Y | N | Y | 60% |
| 24 | Langlois et al., 2020.<br>Adversity in childhood/adolescence and premorbid tobacco, alcohol, and cannabis use among first□episode psychosis patients. | Quantitative Descriptive | Y | N | Y | N | Y | 60% |
| 25 | Li et al., 2011.<br>Longitudinal treatment outcome of African American and Caucasian patients with first episode psychosis. | Quantitative Non-Randomized Controlled Trial | Y | N | Y | N | Y | 60% |
| 26 | Nagendra et al., 2018<br>Demographic, psychosocial, clinical, and neurocognitive baseline characteristics of Black Americans in the RAISE-ETP study. | Quantitative Randomized Controlled Trial | Y | Y | Y | N | Y | 80% |
| 27 | Ramsay et al., 2011<br>Prevalence and psychosocial correlates of prior incarcerations in an urban, predominantly African-American sample of hospitalized patients with first-episode psychosis. | Quantitative Descriptive | Y | N | Y | N | Y | 60% |
| 28 | Ramsay et al., 2011<br>Clinical correlates of maltreatment and traumatic experiences in childhood and adolescence among predominantly African American, socially disadvantaged, hospitalized, first-episode psychosis patients. | Quantitative Descriptive | Y | N | Y | N | Y | 60% |
